# Economic burden of caregiving for persons with severe mental illness in sub-Saharan Africa: A systematic review

**DOI:** 10.1371/journal.pone.0199830

**Published:** 2018-08-09

**Authors:** Rebecca Addo, Samuel Agyei Agyemang, Yesim Tozan, Justice Nonvignon

**Affiliations:** 1 Centre for Health Economics Research and Evaluation (CHERE), University of Technology Sydney, Australia; 2 Department of Health Policy Planning and Monitoring, School of Public Health, University of Ghana, Legon -Accra, Ghana; 3 College of Global Public health, New York University, New York, United States of America; 4 Health Economics, Systems and Policy Research Group, University of Ghana, Legon–Accra, Ghana; West Chester University of Pennsylvania, UNITED STATES

## Abstract

**Background:**

Over the past two decades, the focus of mental health care has shifted from institutionalisation to community-based programs and short hospital stays. This change means that there is an increased role for caregivers, mostly family members, in managing persons with mental illness. Although there is evidence to support the benefits of deinstitutionalisation of mental health care, there are also indications of substantial burden experienced by caregivers; the evidence of which is limited in sub-Saharan Africa. However, knowledge of the nature and extent of this burden can inform the planning of mental health services that will not only benefit patients, but also caregivers and households.

**Objective:**

To systematically review the available evidence on the economic burden of severe mental illness on primary family caregivers in sub-Saharan Africa.

**Methods:**

A comprehensive search was conducted in Pubmed, CINAHL, Econlit and Web of Science with no date limitations up to September 2016 using keywords such as "burden", "cost of illness" and "economic burden" to identify relevant published literature. Articles were appraised using a standardised data extraction tool covering themes such as physical, psychological and socioeconomic burden.

**Results:**

Seven papers were included in the review. Caregivers were mostly family members with a mean age of 46.34, female and unemployed. Five out of seven studies (71%) estimated the full economic burden of severe mental illness on caregivers. The remainder of studies just described the caregiver burden. All seven papers reported moderate to severe caregiver burden characterised by financial constraint, productivity loss and lost employment. The caregiver’s level of income and employment status, severity of patient's condition and duration of mental illness were reported to negatively affect the economic burden experienced by caregivers.

**Conclusion:**

There is paucity of studies reporting the burden of severe mental illness on caregivers in sub-Saharan Africa. Further research is needed to present the nature and extent of this burden to inform service planning and policymaking.

## Introduction

Mental illness, a major public health problem worldwide, refers to any condition that significantly affects the cognition, behaviour, perception and emotions of the affected person. It also affects how the affected person interacts with other people [1, 2]. An individual is said to have severe mental illness when he/she experience serious functional and role impairment with resultant work disability [3]. Examples include depression, schizophrenia, anxiety disorders, bipolar and affective disorder. Recent estimates of the global burden of mental illness have been put around 32·4% of years lived with disability (YLDs) and 13·0% of disability-adjusted life-years (DALYs) globally [4], notably higher than previous estimates of 21.2% of YLDs and 7.1% of DALYs [5]. In sub-Saharan Africa (SSA), mental illness accounts for 19% of YLDs regionally [6]. These estimates place mental illness as one of the leading causes of ill-health and disability.

Over the past two decades, the deinstitutionalisation of mental health care has shifted the focus of the management of people with mental illness from state institutions to homes, implying that family caregivers, who are often unremunerated, take on an increased role in daily care instead of mental health professionals [7]. Studies have shown that caring for a mentally ill patient affects various aspects of caregivers’ life, including their quality of life and socio-economic status [8]. Family caregivers, for instance, are usually required to provide financial support, and endure the burden of economic difficulties. They also provide physical and emotional support to the patient and bear emotional and physical stress resulting from patients disturbing behaviours that consequently affect daily routines and ability to undertake usual social activities [9].

Although there is evidence in support of the benefits of the deinstitutionalization of mental healthcare [10, 11], a growing number of studies has reported on the enormous social and economic burden experienced by primary family caregivers of persons with severe mental illness [12–16]. That said, it is worthy to note that the evidence on the burden on primary family caregivers is limited in SSA. However, the nature and extent of this burden can inform the planning of mental health services that benefits not only patients, but also their caregivers and families.

Studies that had previously reported the economic burden on primary caregivers, either as a review paper or in a systematic review, used different methodological approaches that often did not distinguish between direct costs (medical and non-medical) and indirect costs (income/productivity losses) of these diseases on caregivers and excluded intangible costs. [14, 17, 18]. They mostly adopted a purely descriptive approach to identifying the effects of caregiving on the quality of life of caregivers. However, quantifying the full economic burden associated with a particular disease is key to formulating and prioritizing healthcare policies and interventions [19–21]. The objective of this study was to undertake a systematic review of the evidence on the economic burden of severe mental illness on primary family caregivers in SSA. Compared with previous reviews, this study also provides a description of the direct costs, indirect costs borne by primary family caregivers, and the factors affecting the reported burden.

## Materials and methods

### Search strategy and data sources

A systematic literature search was performed to search for peer-reviewed studies that tried to quantify the economic burden (direct, indirect and intangible costs) on caregivers of persons with severe mental disability in SSA with no date limitations up to September 2016. Searches were conducted in three major electronic databases (PubMed, CINAHL, EconLit and Web of Science) in 2016 from August to September to identify peer-reviewed publications, reports, and working papers using the following keywords: “economic burden”, “cost of illness”, “quality of life”, “caregiver”, “sub-Saharan Africa” and “severe disability”. For the purposes of this study, individuals were considered to have severe mental illness if diagnosed with Dementia, Alzheimer, Depression, Bipolar Disorder, Affective Disorder and Schizophrenia. The search strategies were limited to English language only. A full overview of the electronic search strategies used for different databases is provided as supplementary data (S1 Table). The multiple database searches were stored in EndNote X7 (Thompson Reuters, CA, USA). A full review protocol is available from PROSPERO (PROSPERO 2017: CRD42016051873).

### Inclusion and exclusion criteria

**Fig 1:**
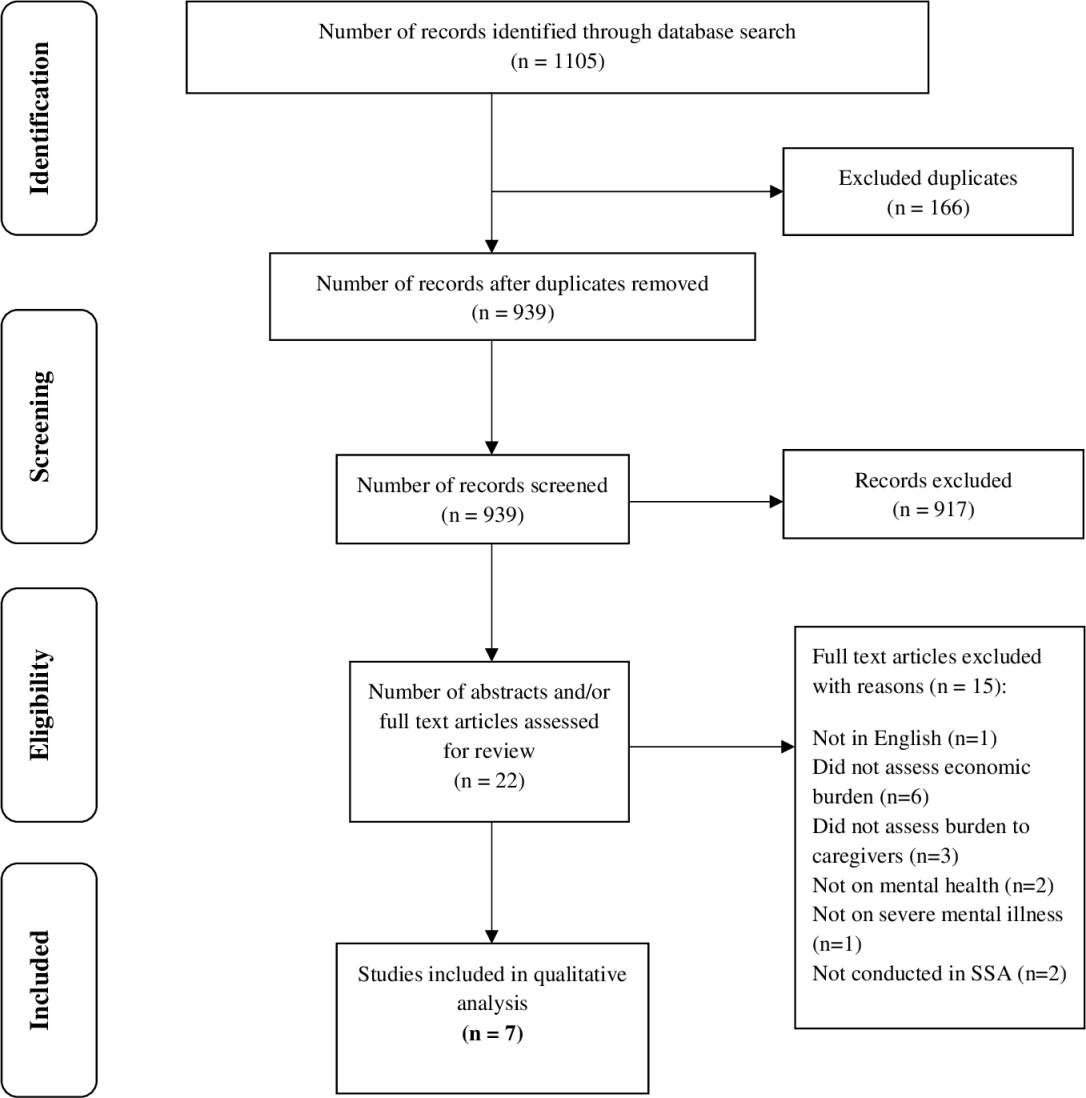
Flow diagram illustrating the steps involved in the review process.

All studies identified were screened for relevance based on predefined inclusion and exclusion criteria. The inclusion criteria were: 1) studies focused on the economic burden on primary family caregivers of people with severe mental illness; 2) studies reported on at least one of the mental illnesses defined as severe in this review; 3) studies with full text accessible; 4) studies with SSA as a setting and 5) studies published in English. Studies were excluded because they were full economic evaluation studies as defined by Drummond et al.[22] These studies include cost effectiveness analysis, cost utility analysis and cost benefit analysis. Seven (n = 7) peer reviewed articles met these criteria and were included in the final review (Fig 1). Two authors independently screened all identified studies based on the title and abstract using the above-mentioned criteria. Disagreements were resolved through discussion after a review of the full-text of all potentially relevant studies. Authors used the Preferred Reporting Items for Systematic Reviews and Meta-Analysis (PRISMA) guidelines and diagram as a guide in identifying relevant studies for the review. A PRISMA flow chart illustrating the selection process is shown in S2 Table

### Data extraction and analysis

Data were extracted independently by two authors. Extracted data were discussed and discrepancies were resolved before final compilation of the results. For consistency in data extraction and subsequent reporting, authors developed a data extraction tool that was used to extract data from all eligible studies. Data extracted include information on study setting, study population and sample size, type of disease, study design, instrument/scale for measuring burden, characteristics of caregivers, direct costs, indirect costs, intangible costs, socioeconomic domain, overall burden rating methods and analysis, factors affecting burden of caregiving, and other relevant information. Extracted data were then entered and analysed in Microsoft Excel 2016. We employed qualitative methods in synthesising the data extracted. The extracted data were critically appraised qualitatively under two main headings: the characteristics of studies and the economic burden of caregivers of persons with severe mental illness.

Cost of illness studies (COIs) present the economic burden of an illness and can be conducted from a number of perspectives such as the patient, household, health systems and societal. Clabaugh and Ward [23] argues that COIs provide policy makers with information on the relative importance of diseases to inform decisions on health priority setting among others. COIs focus on three types of costs; direct, indirect, and intangible [24]. Direct costs include medical costs and non-medical costs incurred due to an illness. Medical costs are the medical care expenditures for diagnosis, treatment, and rehabilitation, while non-medical costs are related to the consumption of non-healthcare resources, such as transportation, food, accommodation, household expenditures, relocation, and property losses, while seeking care. [24]. Indirect costs are costs for which resources are lost, but no direct payment is truly made. They can be classified into two groups: morbidity costs, which are mainly productivity losses borne by the individual and their family, employer, and society as a result of illness; and mortality costs, which are the present value of lost production owing to premature death ensuing from illness [25]. Therefore, we examined the economic burden as direct and indirect costs incurred by primary family caregivers’ due to an illness of a family member; in this instance, severe mental illness. However, it is worth noting that, studies included in this review as to reporting the economic burden of caregiving are those that reported the direct costs only or indirect costs only or both direct and indirect costs or direct. Those that reported only intangible cost that represents mainly psychosocial burden are not included in this review. The factors affecting the extent of this burden were also described.

## Results

The systematic search identified 1105 papers; after the deletion of duplicates, 939 remained of which 917 were excluded after initial screening because they did not satisfy the inclusion criteria of the study. Studies that did not address mental illness but that were conducted in a SSA country were also excluded. Of the 22 studies whose full-text articles were assessed for eligibility, seven (n = 7) were included for the review. These studies were conducted in either a single SSA country or multiple countries, including a SSA country. Fifteen studies were excluded because they were not in English (n = 1), did not report the economic burden (direct and indirect costs) of caregiving (n = 6), did not assess the burden of caregivers (n = 3), did not address mental illness (n = 2), did not address severe disability as defined by this review (n = 1) and studies not conducted in SSA (n = 2).

### Characteristics of studies

Six (86%) studies out of seven used a quantitative study design using a questionnaire, which was a caregiver burden tool either adapted for use or newly developed to capture the different aspects of caregivers’ burden. The remaining study employed qualitative approach (in-depth interviews) to elicit the burden of caregivers using a semi-structured interview guide, with caregiver burden presented in a descriptive manner. Three (43%) of studies were conducted in Nigeria and one each in Ethiopia, Ghana, Zimbabwe and South Africa. Caregivers were recruited mainly at psychiatric health facilities, with sample size ranging from eight [26] to 191 [12] caregivers, as shown in Table 1. Twenty nine percent (29%) of the studies used a caregiver burden scale: either a Zarit Burden Instrument (ZBI), Global Health Questionnaire (GHQ-12) or both [27, 28].

**Table 1:** Characteristics of studies reviewed.

Study	Country of study	Study design	Instrument for measuring burden	Study setting	Sample size	*Characteristics of caregivers	Type of mental illness
Suleiman, Ohaeri, Lawal, Haruna, Orija (1997) [32]	Nigeria	Quantitative	• Semi-structured interview guide with questions on patients and caregivers’ experiences.	Psychiatric out-patient clinic	50	• 44% were males. • Mean age of 42.9 years • 44% of respondents not married, 56% achieved at least secondary level education • 44% were unemployed.	Schizophrenia
Ohaeri (2001) [27]	Nigeria	Quantitative	• A burden questionnaire designed by authors • General Health Questionnaire (GHQ-12)	Psychiatric out-patient clinic	95	• Mean age of 46.7 years for men and 47.3 years for women • 18% (both male and female) were unemployed • 56% were male, and 44% females	Schizophrenia and Major Affective Disorders
Nyati and Sebit (2002) [30]	Zimbabwe	Quantitative	• Standardised questionnaire capturing information on problems faced and costs incurred by caregivers and community perception	Rehabilitation centres, community day centres, resettlement villages, psychiatric units in 3 provinces	66	• Mean age of 48.8 years • 94% of females were unemployed • 20% were males and 80% were females • 78% had either no education or primary education • 36% of caregivers were unemployed	Any form of mental illness, including schizophrenia, bipolar disorders and depression
Prince M. (2004) [29]	15 countries including Nigeria)	Quantitative	• General health questionnaire (GHQ-12) instrument • Zarit burden instrument (ZBI) • Economic impact was assessed using client service receipt inventory	Identification from general population and snowballing	706 in total but only 20 from Nigeria	• 70% were aged 40–64 years., and 30% were <40 years • 50% were unemployed • 5% were males and 95% females • Caregivers were mostly spouses (45% wives) or child (45%) of the patient	Dementia
Zergaw, Hailemariam, Alem and Kebed (2008) [31]	Ethiopia	Quantitative	Questionnaire measuring the longitudinal burden measurement using out-of-pocket medical expenses for health services, time lost due to caregiving and the extent of family caregiving burden.	Homes of participants	190	• 40% were males, and 60% females. • Mean age of 37.58 years with mean family size of 6.23. • Over 80% of the respondents were married. • More than 60% were illiterates with about being housewives • 85% were parents of the patient	Bipolar Disorder
Mavundla, Toth and Mphelane (2009) [26]	South Africa	Qualitative	Semi-structured interview guide with questions on caregivers’ experiences with caregiving	Recruitment done at community clinics, but interviews conducted at Homes of participants	8	• Mean age of 56.9 years • 12% were males, and 88% females • 75% were parents of the patient. The remaining 25% were either a wife or a sister	Any form of mental illness schizophrenia, and bipolar disorders
Addo, Nonvignon and Aikins (2013) [12]	Ghana	Quantitative	A semi-structured questionnaire with questions on the direct, indirect and intangible costs of household members who were caregivers	Psychiatric out-patient clinic	191	• 40% were males, and 60% females • Most were within 20–39 age cohort. • Over 70% of the respondents were employed.	Any form of mental illness schizophrenia, and depression

Footnote

*Characteristics of each study is presented as provided in the individual studies reviewed, hence the differences seen in the categories of characteristics reported

Severe mental illnesses evaluated were dementia [29], bipolar disorder [26, 30, 31], schizophrenia [12, 26, 27, 30, 32], affective disorder [27] and depression [12, 30]. Caregivers interviewed were mainly female and unemployed and had primary education or less. Those who were employed were usually self-employed, mainly farmers and petty traders. Caregivers were on average 40 years and above (mean of 46.34, SD 2.77); close family members, and usually a parent or sibling.

### Economic burden

Table 2 presents a description of how studies reviewed reported the economic burden incurred by caregivers. Five out of seven (71%) of studies reviewed described the component of direct and indirect cost and quantified them in either monetary terms or number of days lost in the case of indirect costs [12, 29–32]. (For the purposes of full disclosure, we acknowledge that the study cited [12] was conducted by the first and last author of the current paper). Two (29%) described only what constituted direct and indirect cost. It is worth noting that one of these studies used qualitative in-depth interviews as a data collection method [26]. Hence, as reported by authors, caregivers gave an account of spending money to seek care and losing some days of work for caregiving and travelling to seek care for relatives (see Table 3). The reason for the other study not quantifying the direct and indirect cost even though it was a quantitative study remains unclear.

**Table 2:** Components of economic burden assessed and reported by reviewed studies.

	Direct costs		Indirect costs	
	Described	Quantified	Described	Quantified
Suleiman, Ohaeri, Lawal, Haruna, Orija (1997) [32]	√	√	√	√
Ohaeri (2001) [27]	√		√	
Nyati and Sebit (2002) [30]	√	√	√	√
Prince M. (2004) [29]	√	√	√	√
Zergaw, Hailemariam, Alem and Kebed (2008) [31]	√	√	√	√
Mavundla, Toth and Mphelane (2009) [26]	√		√	
Addo, Nonvignon and Aikins (2013) [12]	√	√	√	√

Footnote: √ = Described or quantified where described means acknowledging the cost and defining what it constitute and quantified means reporting the amount either as the number of days lost like for indirect cost and/or estimating it in monetary terms).

**Table 3:** Economic burden of caregivers of severe mentally ill patients.

Study	Direct costs	Indirect costs	Factors affecting burden
Suleiman, Ohaeri, Lawal, Haruna, Orija (1997) [32]	• Mean total cost of schizophrenia in six months was US$ 35.9 • The cost of antipsychotic drugs accounts for 52.8% of the total cost	• Relative's loss of working days ranged from 5 to 8 days. • Caregivers lost earnings estimated at US$2.5 per person during the six month period	The purchasing price of drugs was a significant predictor of total cost of illness
Ohaeri (2001) [27]	• *2. 23.2% family took moderate/major loan or sold property to sustain caregiving	• *1. 37.9% of caregivers reported loss of revenue to direct care for patient	• Caregivers of patients who were separated/ divorced experienced a higher and routine family burden and financial distress than caregivers of patients who were married. Same applied to global rating of difficulty with caring for the patient • Caregivers who were unemployed experienced significantly higher financial burden, subjective burden, and GHQ-12 scores
Nyati and Sebit (2002) [30]	• Mean hospital costs 0.01 USD Transportation costs 0.05 USD	• Time spent for care giving ranged 0–3 hours per day • Mean travel time– 107mins (SD 83.8mins)	Not assessed
Prince M. (2004) [29]	• Median healthcare costs (services, medicines and transportation) of 14.1USD • Monies paid to professional careers for caregiving (10% of caregivers)	• Hours spent in informal support per week ranged from none (45%) to 1-10hrs (30%) and 11+hrs (25%) • Median hours per day spent with patient was 4 • Median hours spent per day assisting with activities of daily living was 9 (6–12)	Characteristics of patients such as number of behavioural symptoms and clinical dementia ratings highly correlated with the time spent in caregiving (assisting with activities of daily living)
Zergaw, Hailemariam, Alem and Kebed (2008) [31]	Mean annual out-of-pocket direct medical expenses of $93.93	Family caregivers lost 1.78 days of work due to caregiving	Duration of illness.
Mavundla, Toth and Mphelane (2009) [26]	• *Caregivers reported monies spent on food, transportation, providing shelter and buying medicines	• *Productivity loss to caregiving • *Travel time to seek care • *Inability to sustain regular jobs	Not assessed
Addo, Nonvignon and Aikins (2013) [12]	• Average household cost of mental healthcare per patient per month was US$ 160 • Direct cost included medical (drugs and consultation fees) and non-medical costs (transportation, food, hiring of career and accommodation during admission)	• Average indirect cost 133.31USD • Indirect cost constituted productivity loss due to caregiving, lost employment, travel time, and waiting time	Not assessed

*Direct or indirect costs were only described, but not quantified in monetary terms

GHQ-12 = General Health Questionnaire item 12-scale, HADS = Hospital Anxiety and Depression Scale, ZBI = Zarit Burden Instrument

Table 3 presents a detailed description of the economic burden as reported by studies, and the factors affecting them. Only four (57%) out of seven studies examined the factors that affected the burden experienced by caregivers, and these included duration of illness, severity of symptoms, and socio-economic status of both caregivers and patients. The burden of caregiving was positively associated with these factors, as further explained in Table 3. For instance, as stated by Prince [29], caregivers were likely to spend more productive time to care for persons with severe and debilitating symptoms.

## Discussion

This review focused on the economic burden of caregivers of persons with severe mental illness in SSA countries only. It is the first to be conducted for SSA and provides an overview of the economic burden experienced by caregivers of severely mentally ill persons. This is especially important, as very little study has been conducted in the area of mental health in the region, most especially the burden it confers on caregivers. Meanwhile, unlike most developed countries, there are no rehabilitation centres and other supportive services tailored for people living with mental illness, hence family members, who are primary caregivers, bear the responsibility of caring for patients who are not institutionalised. Therefore, we conducted this review to collate studies in this area for easy access by policy makers who may not have the time or resources to systematically search for them, for possible input into policymaking, when needed. In addition, the review sought to report the quantity of studies available in this area to inform future research. Hence, the review excludes results from other parts of the world.

While 71% (n = 5) of papers reviewed in the current study described and quantified both direct and indirect costs due to caregiving, the remaining two (29%) studies described them. Socio-economic characteristics of caregivers such as employment status and income level, and patient characteristics, such severity of symptoms and duration of illness, positively affected the economic burden reported by caregivers. The study searched four different databases for published literature.

Other studies assessing the burden (which includes economic) on informal caregivers of any form of mental illness either among SSA population [18] or those from other parts of the world caregivers [17, 26, 27, 29, 30, 33, 34], who concentrated on the effect of caregiving on the quality of life of caregivers. These studies used a scale measuring the psychological and physical aspects of caregiving that contributed to the burden experienced by caregivers. This methodological approach of estimating the burden of mental illness can explain the use of burden scales by two (29%) of the papers reviewed in the current study. Just as the quality of life affects every dimension of the caregivers’ life, other forms of economic burden; direct costs and indirect costs also affects the quality of life of the caregiver either directly or indirectly.

For instance, while the psychological burden of caregiving was reported by some studies conducted in SSA to be higher among patients with long duration of illness and severe and debilitating symptoms [28, 35, 36], the current review also showed that these illness characteristics were also associated with higher productivity losses as caregivers tended to spend more time caring for such patients [29, 31]. Therefore, the indirect costs in the form of productivity losses constituted the largest portion of the total economic burden, as reported by Addo et al. [12]. Our findings also corroborate that of Sado et al. et al. [37] in their review of the burden of schizophrenia conducted among Japanese population, and Pratima et al. [34] who assessed the burden of severe mental illness among caregivers in Delhi, India. This study further affirms a review by Fajutrao et al [38] on indirect costs (lost productivity in particular) being a major contributor to the burden of bipolar disorder in Europe.

We further found that 17–50% of caregivers of persons with severe mental illness were unemployed. It is worth noting that, although, the methodological approach used in this study cannot establish a causal relationship between unemployment and caregiving, evidence from the papers reviewed provided a possible association between these two variables. This is evidenced by a majority of caregivers reporting inability to work either full time or part-time due to their caregiving responsibilities [27, 29, 30]. Those doing any form of work also reported that they had to cut back on the number of working hours [12, 26]. Indeed, Bauer and Sousa-Poza in their discussion paper that reviewed a number of studies investigating the relationship between informal caregiving and lower levels of employment concluded that, there was an association between these two variables, even though the affected labour force is small [33]. That said, further research need to be conducted to understand how caregiving responsibilities affect household income and productivity among those caring for the severely mentally ill, as these conditions are chronic and tend to be debilitating in most instances, hence the extent of caregiving is expected to vary compared to that of the general population.

Productivity losses due to caregiving is a major economic burden on both the caregiver and the society as a whole, hence must not be overlooked. Unfortunately, majority (86%) of studies reviewed did not estimate this portion of economic burden monetary terms [26, 27, 29–32]. This is a major limitation of these studies. Another shortfall of two (29%) of these studies is that they failed to estimate the direct costs incurred by caregivers in caring for their family members, such as financial resources expended in the form of transportation, cost of drugs, cost of consultations and hospitalizations among others.

The importance of this burden was presented by an earlier study which reported that 23% of families of persons with schizophrenia and affective disorders resorted to either selling their property or taking a moderate to mild loan to be able to continue seeking care for their relatives [27]. In our review, only Addo et al [12] and Nyati and Sebit [30] presented the direct medical and non-medical costs borne by primary caregivers of mentally ill patients in monetary terms. In another study that described the burden of mental illness in Ghana, caregivers reported selling their property in the effort to take care of their relatives who suffered from severe mental illness [8]. As described in this review, Jack-Ide [39] and Allers et al [13] also reported the costs borne by caregivers of patients living with mental illness (epilepsy) in the form of transportation, costs of medication and time spent in seeking care and for caregiving. Aller et al further stressed that these costs might vary according to the severity of the condition, the response to treatment, and the duration of illness, which is similar to what is reported by some studies included in this review.

It is evident that the direct and indirect costs incurred by caregivers is substantial, given the economic context of the study area. For instance, Addo et al [12] report that about 71% of caregivers in Ghana reported monthly income of less than $223, consistent with recent estimate that about 94% of caregivers (of elderly persons) in Ghana reported monthly incomes of less than $224 [40]. Therefore, failure to identify, measure and quantify these costs leads to an underestimation of the economic burden experienced by informal caregivers of persons with severe mental illness. As a result, policy makers are misinformed of the exact burden on informal caregivers when findings from such studies are considered for decision-making, leading to mental health planning that fails to address the needs of caregivers.

The findings of this review are limited because the search strategy was limited to peer-reviewed and published articles in international databases. Unpublished reports, seminar presentations, doctoral and master’s theses and studies from the grey literature were not captured. Furthermore, relevant information published in journals that are not registered online was also not included in the review. The findings of the review might have been biased as they were subject to reviewers’ interpretation. However, this limitation was minimised, as two reviewers used a standardised data extraction tool to extract data independently and resolved discrepancies through discussion.

## Conclusion

In SSA countries, there remains a paucity of literature examining the economic burden for caregivers of persons with severe mental illness. However, for decision makers to make mental health policies that adequately address the needs of the recent paradigm of mental health care delivery, deinstitutionalization, they need to be provided with information of both its benefits and consequences. Some of the consequences of deinstitutionalisation can be established by estimating the burden, including, but not restricted to, quantifying the economic burden of mental healthcare to caregivers.

## Supporting information

S1 Table Search strategy. (PDF)

S2 Table PRISMA 2009 checklist. (DOC)

## References

[1] Manderscheid RW, Ryff CD, Freeman EJ, McKnight-Eily LR, Dhingra S, Strine TW. Evolving definitions of mental illness and wellness. Prev Chronic Dis. 2010;7(1):A19 20040234 PMC2811514

[2] Mental disorders [Internet]. WHO. 2017 [cited 19/05/2017]. Available from: http://www.who.int/mediacentre/factsheets/fs396/en/.

[3] Kessler RC, Chiu WT, Demler O, Walters EE. Prevalence, severity, and comorbidity of 12-month DSM-IV disorders in the National Comorbidity Survey Replication. Archives of general psychiatry. 2005;62(6):617–27. 10.1001/archpsyc.62.6.617 15939839 PMC2847357

[4] Vigo D, Thornicroft G, Atun R. Estimating the true global burden of mental illness. The lancet Psychiatry. 2016;3(2):171–8. Epub 2016/02/07. 10.1016/S2215-0366(15)00505-2 . 26851330

[5] Institute for Health Metrics and Evaluation. The Global Burden of Disease: Generating Evidence, Guiding Policy–European Union and European Free Trade Association Regional Edition. Seattle: IHME, 2013.

[6] Whiteford HA, Degenhardt L, Rehm J, Baxter AJ, Ferrari AJ, Erskine HE, et al Global burden of disease attributable to mental and substance use disorders: findings from the Global Burden of Disease Study 2010. The Lancet. 382(9904):1575–86. 10.1016/S0140-6736(13)61611-6 23993280

[7] Thompson EH, Doll W. The burden of families coping with the mentally ill: An invisible crisis. Family Relations. 1982;31:379–88.

[8] Ae-Ngibise KA, Doku VCK, Asante KP, Owusu-Agyei S. The experience of caregivers of people living with serious mental disorders: a study from rural Ghana. Global Health Action. 2015;8: 10.3402/gha.v8.26957 PMC4429259. 25967587 PMC4429259

[9] World Health Organization. WHO mental health: a call for action by World Health Ministers Ministerial round tables. 54th World Health Assembly. WHO Geneva, Switzerland, 2001.

[10] Emerson E, Hatton C. Deinstitutionalization in the UK and Ireland: Outcomes for service users. Journal of Intellectual & Developmental Disability. 1996;21(1):17–37. 10.1080/13668259600033021

[11] Kliewer SP. Deinstitutionalization: Its Impact on Community Mental Health–ERIC. 2009.

[12] Addo R, Nonvignon J, Aikins M. Household costs of mental health care in Ghana. The journal of mental health policy and economics. 2013;16(4):151–9. Epub 2014/02/15. . 24526584

[13] Allers K, Essue BM, Hackett ML, Muhunthan J, Anderson CS, Pickles K, et al The economic impact of epilepsy: a systematic review. BMC Neurology. 2015;15:245 10.1186/s12883-015-0494-y PMC4660784. 26607561 PMC4660784

[14] Bhimani R. Understanding the Burden on Caregivers of People with Parkinson’s: A Scoping Review of the Literature. Rehabilitation Research and Practice. 2014;2014:8 10.1155/2014/718527 25298895 PMC4179947

[15] Savage S, Bailey S. The impact of caring on caregivers' mental health: a review of the literature. Australian Health Review. 2004;27(1):111–7. 10.1071/AH042710111. 15362303

[16] Vaingankar JA, Chong SA, Abdin E, Picco L, Jeyagurunathan A, Zhang Y, et al Care participation and burden among informal caregivers of older adults with care needs and associations with dementia. International Psychogeriatrics / Ipa. 2016;28(2):221–31. 10.1017/S104161021500160X PMC4720142. 26478530 PMC4720142

[17] Caqueo-Urizar A, Gutierrez-Maldonado J, Miranda-Castillo C. Quality of life in caregivers of patients with schizophrenia: a literature review. Health Qual Life Outcomes. 2009;7:84 Epub 2009/09/15. 10.1186/1477-7525-7-84 ; PubMed Central PMCID: PMCPmc2749816. 19747384 PMC2749816

[18] Esan O, Esan A. Epidemiology and burden of bipolar disorder in Africa: a systematic review of data from Africa. Social Psychiatry and Psychiatric Epidemiology. 2016;51(1):93–100. 10.1007/s00127-015-1091-5 26155900

[19] Costa N, Derumeaux H, Rapp T, Garnault V, Ferlicoq L, Gillette S, et al Methodological considerations in cost of illness studies on Alzheimer disease. Health Economics Review. 2012;2(1):18 10.1186/2191-1991-2-18 22963680 PMC3563616

[20] Kobelt G. Health Economics An Introduction To Economic Evaluation 2 London: Office of Health Economics 2002.

[21] Tarricone R. Cost-of-illness analysis. What room in health economics? Health policy (Amsterdam, Netherlands). 2006;77(1):51–63. Epub 2005/09/06. 10.1016/j.healthpol.2005.07.016 . 16139925

[22] Drummond MF, Sculpher MJ, Claxton K, Stoddart GL, Torrance GW. Methods for the economic evaluation of health care programmes: Oxford university press; 2015.

[23] Clabaugh G, Ward MM. Cost-of-Illness Studies in the United States: A Systematic Review of Methodologies Used for Direct Cost. Value in Health. 2008;11(1):13–21. 10.1111/j.1524-4733.2007.00210.x. 18237356

[24] Jo C. Cost-of-illness studies: concepts, scopes, and methods. Clinical and Molecular Hepatology. 2014;20(4):327–37. 10.3350/cmh.2014.20.4.327 PMC4278062. 25548737 PMC4278062

[25] Cooper NJ. Economic burden of rheumatoid arthritis: a systematic review. Rheumatology. 2000;39(1):28–33. 10.1093/rheumatology/39.1.28 10662870

[26] Mavundla TR, Toth F, Mphelane ML. Caregiver experience in mental illness: a perspective from a rural community in South Africa. International journal of mental health nursing. 2009;18(5):357–67. Epub 2009/09/11. 10.1111/j.1447-0349.2009.00624.x . 19740145

[27] Ohaeri JU. Caregiver burden and psychotic patients' perception of social support in a Nigerian setting. Soc Psychiatry Psychiatr Epidemiol. 2001;36(2):86–93. Epub 2001/05/18. . 11355450 10.1007/s001270050294

[28] Shibre T, Kebede D, Alem A, Negash A, Deyassa N, Fekadu A, et al Schizophrenia: illness impact on family members in a traditional society—rural Ethiopia. Soc Psychiatry Psychiatr Epidemiol. 2003;38(1):27–34. Epub 2003/02/04. 10.1007/s00127-003-0594-7 . 12563556

[29] Prince M. Care arrangements for people with dementia in developing countries. International journal of geriatric psychiatry. 2004;19(2):170–7. Epub 2004/02/06. 10.1002/gps.1046 . 14758582

[30] Nyati Z, Sebit MB. Burden of mental illness on family members, care-givers and the community. East African medical journal. 2002;79(4):206–9. Epub 2003/03/11. . 12625678 10.4314/eamj.v79i4.8880

[31] Zergaw A, Hailemariam D, Alem A, Kebede D. A longitudinal comparative analysis of economic and family caregiver burden due to bipolar disorder. African journal of psychiatry. 2008;11(3):191–8. Epub 2009/07/10. . 19588042 10.4314/ajpsy.v11i3.30268

[32] Suleiman TG, Ohaeri JU, Lawal RA, Haruna AY, Orija OB. Financial cost of treating out-patients with schizophrenia in Nigeria. The British journal of psychiatry: the journal of mental science. 1997;171:364–8. Epub 1997/11/28. . 9373427 10.1192/bjp.171.4.364

[33] Bauer JM, Sousa-Poza A. Impacts of Informal Caregiving on Caregiver Employment, Health, and Family. IZA Discussion Paper. 2015;(8851).

[34] Pratima, Bhatia MS, Jena SPK. Caregiver burden in severe mental illness. Delhi Psychiatry J 2011;14:211–9.

[35] Tajudeen Nuhu F, Jika Yusuf A, Akinbiyi A, Oluyinka Fawole J, Joseph Babalola O, Titilope Sulaiman Z, et al The burden experienced by family caregivers of patients with epilepsy attending the government psychiatric hospital, Kaduna, Nigeria. The Pan African Medical Journal. 2010;5:16 PMC3032618. 21293743 10.4314/pamj.v5i1.56176 PMC3032618

[36] Yusuf AJ, Nuhu FT. Factors associated with emotional distress among caregivers of patients with schizophrenia in Katsina, Nigeria. Soc Psychiatry Psychiatr Epidemiol. 2011;46(1):11–6. Epub 2009/11/13. 10.1007/s00127-009-0166-6 . 19907909

[37] Sado M, Inagaki A, Koreki A, Knapp M, Kissane LA, Mimura M, et al The cost of schizophrenia in Japan. Neuropsychiatric disease and treatment. 2013;9:787–98. Epub 2013/06/21. 10.2147/NDT.S41632 ; PubMed Central PMCID: PMCPmc3682806. 23785238 PMC3682806

[38] Fajutrao L, Locklear J, Priaulx J, Heyes A. A systematic review of the evidence of the burden of bipolar disorder in Europe. Clinical Practice and Epidemiology in Mental Health. 2009;5(1):3 10.1186/1745-0179-5-3 19166608 PMC2646705

[39] Jack-Ide IO, Uys LR, Middleton LE. Caregiving experiences of families of persons with serious mental health problems in the Niger Delta region of Nigeria. International journal of mental health nursing. 2013;22(2):170–9. Epub 2012/06/21. 10.1111/j.1447-0349.2012.00853.x . 22712889

[40] Nortey ST, Aryeetey GC, Aikins M, Amendah D, Nonvignon J. Economic burden of family caregiving for elderly population in southern Ghana: the case of a peri-urban district. International Journal for Equity in Health. 2017;16:16 10.1186/s12939-016-0511-9 PMC5237474. 28088236 PMC5237474

