# Supplementary material for: Economic burden of caregiving for persons with severe mental illness in sub-Saharan Africa: A systematic review
Source: PLoS One. 2018 Aug 9;13(8):e0199830. doi: 10.1371/journal.pone.0199830 (PMC6084810; doi:10.1371/journal.pone.0199830)
Supplement: S1 Table — (PDF) [file pone.0199830.s001.pdf]

Domain	Sub-category	Search	Search terms<br>Pubmed
Economic burden	Direct healthcare cost	1	Cost OR economic OR “cost of illness” OR “burden of illness” OR “illness burden” OR “economic burden” OR expenditure OR “quality of life” OR funding
Caregivers of patients	Caregivers	2	Caregivers OR carer OR caregiver*OR "family member" OR spouse OR partner OR [illegible]
Severe disability	Dementia	3	“dementia”[MeSH Terms] OR “dementia”[All Fields]”
	Alzheimer	4	“alzheimer* or exp *Alzheimer disease/”
	Bipolar Disorder	5	“bipolar disorder” OR bipolar OR mania OR manic
	Depression	6	Depression [MeSH Terms] OR Depression [All Fields] OR Depresive disorder [MeSH]
	Schizophrenia	7	“Schizophrenia”[MeSH Terms] OR “Schizophrenia”[All Fields]
Sub-Saharan Africa		8	(Africa OR African OR "Africa south of the Sahara” OR Angola OR Benin OR Botswana OR Burkina Faso OR Burundi OR Cameroon OR Cape Verde OR Central African Republic OR Chad OR Comoros OR Congo OR Cote d'Ivoire OR Djibouti OR Eritrea OR Ethiopia OR Gabon OR Gambia OR Ghana OR Guinea OR Kenya OR Lesotho OR Liberia OR Madagascar OR Malawi OR Mali OR Mauritania OR Mauritius OR Mozambique OR Namibia OR Niger OR Nigeria OR Rwanda OR Sao Tome OR Principe OR Senegal OR Seychelles OR Sierra Leone OR Somalia OR South Africa OR Sudan OR Swaziland OR Tanzania OR Togo OR Uganda OR Zambia OR Zimbabwe)
Combined search in titles, abstracts and keywords			#1 AND #2 AND (#3 OR #4 OR #5 OR #6 OR #7 OR #8)
